# Strong Photo-Amplification Effects in Flexible Organic Capacitors with Small Molecular Solid-State Electrolyte Layers Sandwiched between Photo-Sensitive Conjugated Polymer Nanolayers

**DOI:** 10.1038/srep19527

**Published:** 2016-02-05

**Authors:** Hyena Lee, Jungnam Kim, Hwajeong Kim, Youngkyoo Kim

**Affiliations:** 1 Organic Nanoelectronics Laboratory, Department of Chemical Engineering, School of Applied Chemical Engineering, Kyungpook National University, Daegu 702-701, Republic of Korea; 2 Priority Research Center, Research Institute of Advanced Energy Technology, Kyungpook National University, Daegu 702-701, Republic of Korea

## Abstract

We demonstrate strong photo-amplification effects in flexible organic capacitors which consist of small molecular solid-state electrolyte layers sandwiched between light-sensitive conjugated polymer nanolayers. The small molecular electrolyte layers were prepared from aqueous solutions of tris(8-hydroxyquinoline-5-sulfonic acid) aluminum (ALQSA_3_), while poly(3-hexylthiophene) (P3HT) was employed as the light-sensitive polymer nanolayer that is spin-coated on the indium-tin oxide (ITO)-coated poly(ethylene terephthalate) (PET) film substrates. The resulting capacitors feature a multilayer device structure of PET/ITO/P3HT/ALQSA_3_/P3HT/ITO/PET, which were mechanically robust due to good adhesion between the ALQSA_3_ layers and the P3HT nanolayers. Results showed that the specific capacitance was increased by ca. 3-fold when a white light was illuminated to the flexible organic multilayer capacitors. In particular, the capacity of charge storage was remarkably (ca. 250-fold) enhanced by a white light illumination in the potentiostatic charge/discharge operation, and the photo-amplification functions were well maintained even after bending for 300 times at a bending angle of 180^o^.

Capacitors have significantly contributed to the development of both energy storage systems on a macroscopic scale and information storage (memory) devices on a microscopic scale^1^,^2^,^3^,^4^,^5^. The energy storage capacitors can be categorized into two types, electrostatic and electrochemical (electrolytic) capacitors, according to the charge storage mechanism^6^,^7^,^8^. A dielectric film plays a core role in charge storage at the interface with electrodes in the case of electrostatic capacitors, while the charge storage in the case of electrochemical capacitors is basically dependent on the ion transport of electrolytes^9^,^10^. In particular, supercapacitors (or ultracapacitors) have been spotlighted due to their high energy density, covering between conventional electrolytic capacitors and secondary (rechargeable) batteries, which can be achieved by introducing sophisticated electrodes with high specific surface area^11^,^12^,^13^,^14^,^15^.

However, conventional electrochemical capacitors including supercapacitors do mostly rely on the liquid-based electrolytes, which are understood a critical disadvantage toward advanced capacitors in the coming flexible electronics era because the presence of liquid-type electrolytes limits the design freedom for ultrathin and highly flexible devices owing to the practical leakage issue of liquid electrolyte components^16^,^17^,^18^,^19^,^20^,^21^,^22^. The reason for using such liquid-based electrolytes can be attributable to the lack of suitable solid-state electrolyte with easy processability, even though a couple of solid-state electrolytes have been proposed^23^,^24^,^25^,^26^,^27^. Interestingly, most of electrode materials in conventional capacitors do not have optical transparency, and the operation of capacitors has been restricted to electrical methods such as galvanostatic (applying constant current only) and potentiostatic (applying constant voltage only) modes.

Very recently, all-solid-state organic capacitors have been reported by introducing a sold-state electrolyte of organic-metal chelate complex, tris(8-hydroxyquinoline-5-sulfonic acid) aluminum (ALQSA_3_), which can be coated from aqueous solutions^28^. In particular, the transparent conducting oxide (TCO)-coated plastic film substrates with high optical transparency could be used for the fabrication of such all-solid-state organic capacitors. Therefore various optical methods can be used for the operation of the capacitors with the TCO-coated substrates because photons are allowed to come into active layers including solid-state electrolytes. Hence this optical approach is considered to open new step for capacitor applications.

In this work, we demonstrate optically-chargeable flexible organic capacitors, in which photosensitive nanolayers take place on both sides of solid-state electrolyte (ALQSA_3_) layer and contact each transparent electrode individually. The photosensitive nanolayers were fabricated using poly(3-hexylthiopene) (P3HT), while indium-tin oxide (ITO)-coated poly(ethylene terephthalate) (PET) films were used as the TCO-coated substrates. The resulting device structure is PET/ITO/P3HT/ALQSA_3_/P3HT/ITO/PET (PIPAPIP). The optical charging effect was examined by galvanostatically and potentiostatically operating flexible organic capacitors under illumination with a simulated solar (white) light (100 mW/cm^2^).

## Results

**Figure 1:**
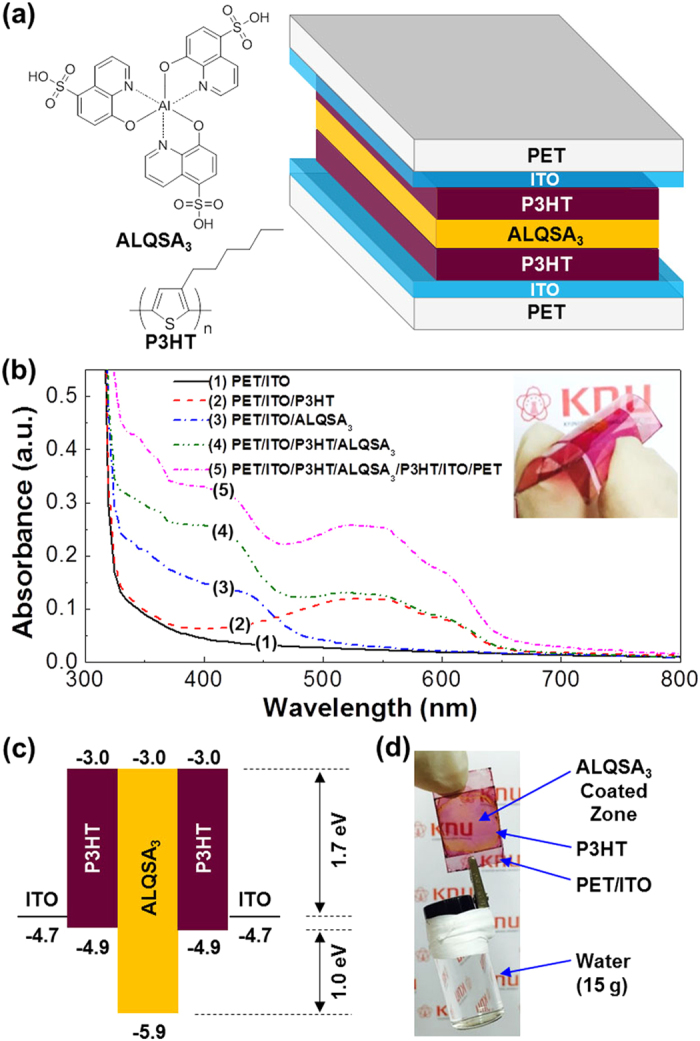
(**a**) Device structure of flexible multilayer capacitors and materials used for the core layers: tris(8-hydroxyquinoline-5-sulfonic acid) aluminum (ALQSA_3_) and poly(3-hexylthiophene) (P3HT). (**b**) Optical absorption spectra for each layer in the flexible capacitors (see inset for the bended device). (**c**) Flat energy band structure for the flexible capacitor (note that the ‘eV’ unit is omitted from the energy values). (**d**) Photograph for the adhesion test between the ALQSA_3_ layer and the P3HT nanolayers: A water bottle (15 g) was suspended to one side of the ITO-PET substrates, while another side was kept by fingers. Note that no slip in the devices was observed after 24 h upon loading of the 15 g water bottle.

As shown in Fig. 1a, the flexible organic capacitors have a multilayer device configuration of active layers sandwiched between two ITO-PET films. Considering the excellent optical transparency of the ITO-PET substrates (see optical absorption spectrum in Fig. 1b), photons (visible lights) can pass through the substrates and reach the active layers (P3HT/ALQSA_3_/P3HT) from both sides of flexible organic capacitors. When a white light (sun light) is illuminated to the present organic capacitors, the P3HT nanolayers (thickness = 50 nm) are optically excited to make excitons that can be separated into individual charges (electrons and holes). These optically generated charges are added to the charges generated by the action of ion transport in the ALQSA_3_ layer under bias condition, which results in synergistic effects in terms of charge storage capacity inside devices. Here we note that the optical amplification effect was negligible for the PET/ITO/ALQSA_3_/ITO/PET device without the P3HT nanolayers because of the limited optical absorption far below 500 nm by the ALQSA_3_ layer (see Fig. 1b).

Taking into account the flat energy band diagram in Fig. 1c, the external electron injection from the ITO electrode to the lowest unoccupied molecular orbital (LUMO) energy level of P3HT and ALQSA_3_ cannot be allowed at less than 1.7 V (bias condition). In contrast, the external hole injection into the highest occupied molecular orbital (HOMO) energy level of P3HT from the ITO electrode may be possible owing to the very small energy barrier (0.2 eV) but the holes will be blocked by the relatively huge HOMO energy barrier (1 eV) with the ALQSA_3_ layer. Hence, the present devices can be operated as a pure capacitor in the dark, which makes energy (current or voltage) storage by internal ion transport in the solid-state electrolyte layer (ALQSA_3_) without external charge injection, by controlling a bias voltage of <1 V. The proper construction of the multilayer structure was confirmed by the excellent adhesion between the ALQSA_3_ layer and the P3HT nanolayers, as supported by no slip upon loading of a water bottle (15 g) on one side of the ITO-PET substrates in the flexible organic capacitors (see Fig. 1d).

**Figure 2:**
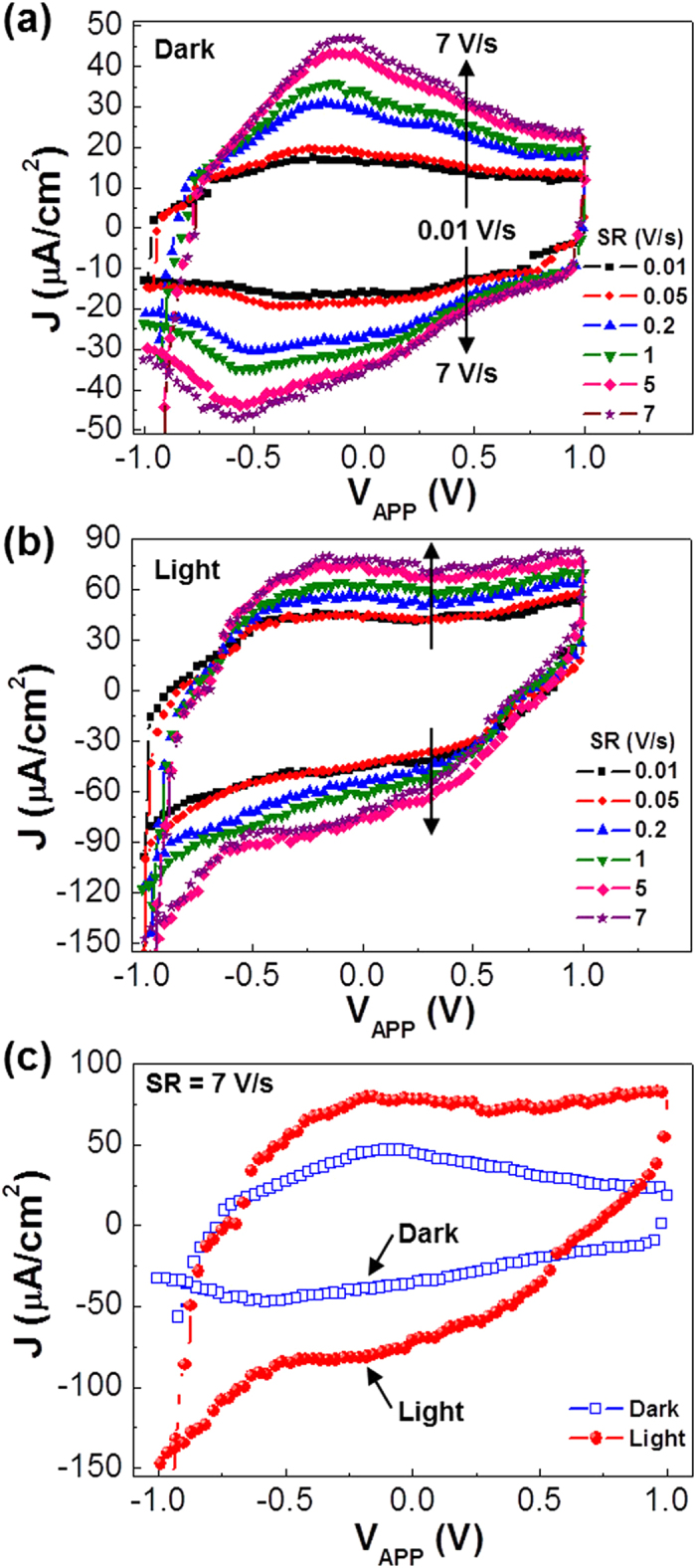
(**a,b**) Cyclic voltammetry (CV) curves for the flexible capacitors according to the voltage scan rate (SR): (**a**) in the dark, (**b**) under illumination with a white light (100 mW/cm^2^). (**c**) Comparison of the CV curve in the dark with that under the white light at SR = 7 V/s.

To examine whether the present flexible organic capacitors do properly work, cyclic voltammetry (CV) tests were performed by varying the voltage scan rate (SR) from 0.01 V/s to 7 V/s. As shown in Fig. 2a, the CV curves in the dark exhibited pronounced hysteresis in current density irrespective of scan rate. In particular, the higher the scan rate, the larger the extent of hysteresis. This result does basically indicate that charges can be stored in the present devices and the amount of charges can be controlled by adjusting the scan rate (note that the increment in current density was slowed down at higher scan rates). Interestingly, as shown in Fig. 2b, the current density in the CV curves was significantly increased but still proportional to the scan rate upon illumination with a white light (light intensity = 100 mW/cm^2^) (see Fig. S1a), even though the shape of CV curves under illumination was slightly changed from that in the dark. Here, a particular attention is paid to the well-maintained hysteresis even under the white light (see Fig. S1b), which supports that the present devices are able to function as a capacitor under sun light. As directly compared in Fig. 2c (SR = 7 V/s), the CV curve under the white light showed much higher current density and hysteresis than that in the dark. To exactly check the contribution of white light illumination to the charge capacity in the present devices, the specific capacitance (C_S_) was calculated using 
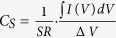
 from the CV curves^29^. The calculated C_S_ was 2.46 mF/cm^2^ in the dark, while it reached 7.58 mF/cm^2^ under the white light. This result reveals that the specific capacitance could be improved by more than 3-fold when the devices were operated under the white light.

**Figure 3:**
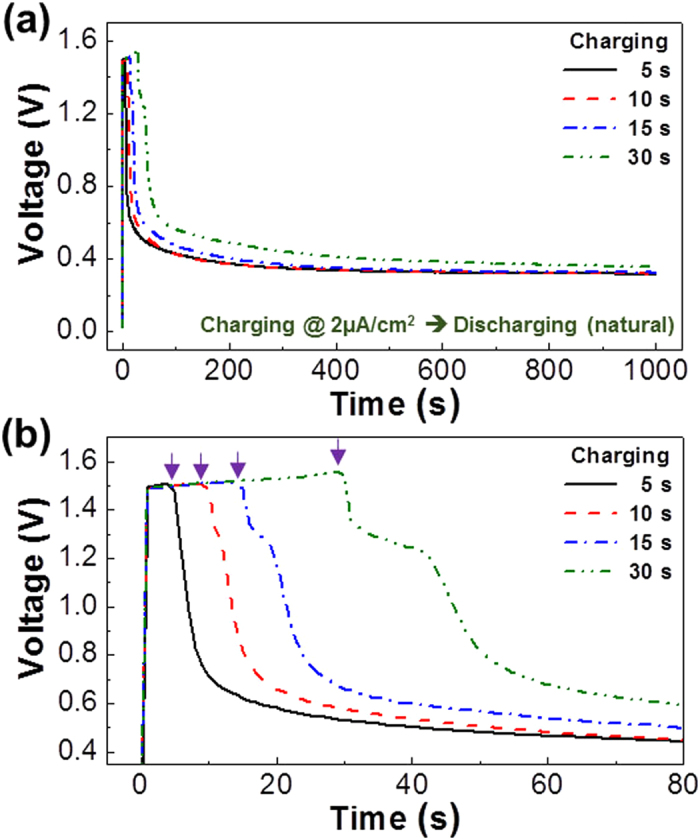
(**a**) Galvanostatic charge/discharge curves for the flexible capacitors at 2 μA/cm^2^ according to the charging time (note that a self-discharge was employed). (**b**) Enlarged plots focusing on the peak parts from (**a**): The arrows denote the end points of charging for each charging time.

Next, the performances of capacitors were measured under galvanostatic (constant current mode) conditions at 2 μA/cm^2^ in order to test whether the present devices exhibit proper charge-discharge characteristics. As shown in Fig. 3a, the device voltage in the dark was quickly increased up to 1.5 V upon applying 2 μA/cm^2^ (charging), which was independant with the charging time (see Fig. 3b). The voltage was well kept at the constant current condition in the presence of marginal increase with the charging time (from 1.5 V to 1.56 V for 30 s). When the constant current was removed (discharging mode), the device voltage was initially quickly decayed and then a slow decay phase was measured in the case of the short time charging (5 s). When the charging time was further increased, an additional voltage bump appeared and its width became wider as the charging time was more increased. The size of this voltage bump may indicate the amount of charges stored at the constant current condition. Here it is worthy to note that the device voltage was still higher than 0.3 V even after 1000 s in the case of 5 s charging (see Fig. 3a) and it became higher as the charging time was more increased (see Fig. 3b). This result reflects that the proton ions transported toward the counter electrode upon charging could not be fully moved back toward the original electrode in the natural discharging condition without any external loads^30^,^31^.

**Figure 4:**
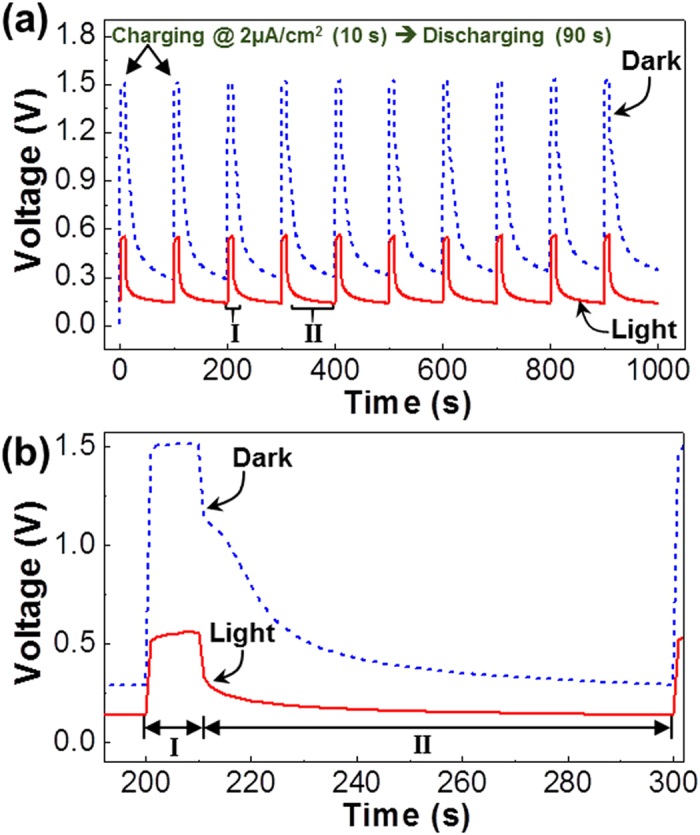
(**a**) Repeated galvanostatic charge/discharge curves for the flexible capacitors at 2 μA/cm^2^ (charging time = 10 s) in the dark and under illumination with a white light (100 mW/cm^2^) (note that a self-discharge was employed). (**b**) Enlarged plots focusing on one representative peak from (**a**).

Based on the above device performances, the galvanostatic charge-discharge characteristics under the white light were compared with those in the dark. As shown in Fig. 4a, a stable voltage pattern with quick increase (up to ca. 1.5 V) and tailed decay responses was measured in the dark upon the repeated charge-discharge operation. The similar quick response was measured for the same devices under the white light but the device voltage reached ca. 0.37 V at the same galvanostatic condition (see Fig. 4b). This reduced voltage can be attributed to the decreased electrical resistance owing to the generation of charges (and excitons) under the white light. Therefore, it is shortly concluded that the white light illumination could adversely affect the output voltage in the case of galvanostatic mode.

**Figure 5:**
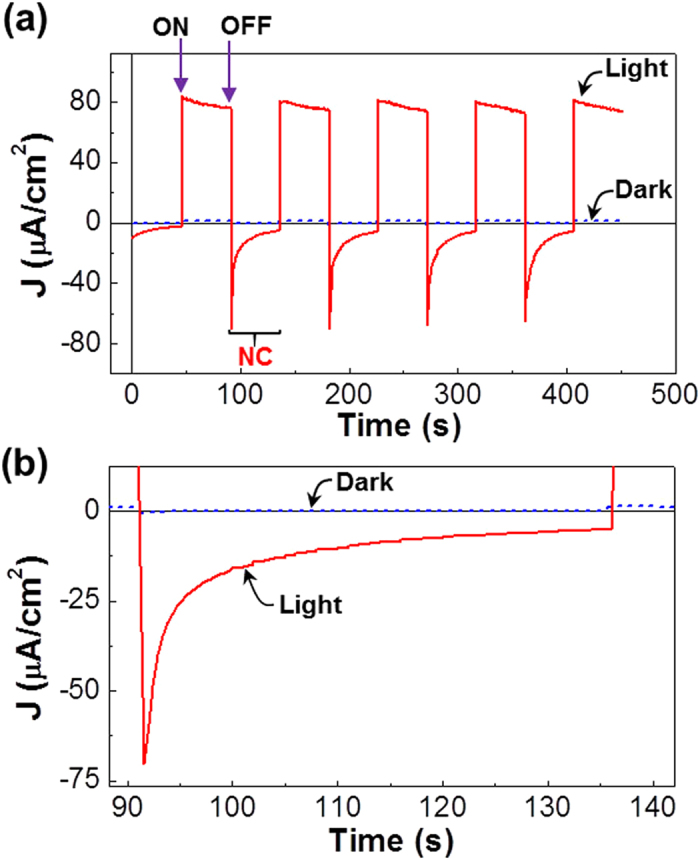
(**a**) Repeated potentiostatic charge/discharge curves for the flexible capacitors in the dark and under illumination with a white light (100 mW/cm^2^): ‘ON’ and ‘OFF’ denote applying +1 V and removing +1 V (i.e., to 0 V), respectively, while ‘NC’ means the negative current part. (**b**) Enlarged plots focusing on one representative NC peak from (**a**).

**Figure 6:**
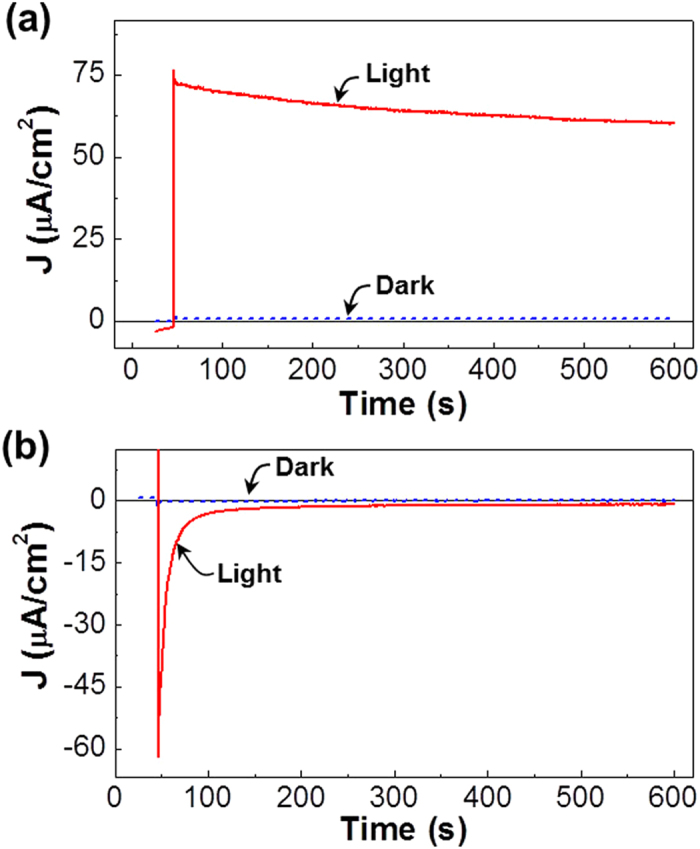
(**a**) ON-state (+1 V) current—time curves for the flexible capacitors in the dark and under illumination with a white light (100 mW/cm^2^) by extending the charging time up to 600 s. (**b**) OFF-state (0 V) current—time curves for the flexible capacitors in the dark and under illumination with a white light (100 mW/cm^2^), which were measured just after switching the applied voltage from +1 V to 0 V (note that a self-discharge was employed).

To further investigate the effect of light illumination, the device performances were examined under potentiostatic (constant voltage) conditions. As shown in Fig. 5a, the device current was quickly increased upon applying +1 V (ON state) and it was kept in the presence of marginal decay during the ON state. Here it is noted that the ON current level was significantly higher under the white light (10 μA) than in the dark (0.2 μA). When the applied voltage was removed (OFF state), the device current was quickly decreased for both dark and light illumination cases (see Fig. S2a for the enlarged graph in the dark). Here, interestingly, the device current did not stop at 0 A after removing the applied voltage (OFF state), but it did immediately overshoot to the negative direction (−8.5 μA for light and −0.08 μA for dark). Furthermore, the negative current underwent a slow decay process after reaching the (negative) maximum values, which was much more pronounced under the white light than in the dark (see Fig. S2b for the enlarged graph in the dark). So this negative current (NC) part can be corresponded to the net charges stored by the potentiostatic charging operation. The amount of charges (Q) stored can be calculated using Q = 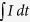 for the area in the negative current part (from 90 s to 137 s in Fig. 5b). The calculated amount of charges was 7 × 10^−5^ C under the white light and 2.9 × 10^−7^ C in the dark, which discloses that the charge storage could be amplified by 250-fold under the white light (see video clips in Fig. S3 for the photo-amplified charging operation). As shown in Fig. 6, the similar remarkable charge storage under the white light could be achieved even in the extended charging/discharging time up to >500 s (see Fig. S4 for the dark case).

**Figure 7:**
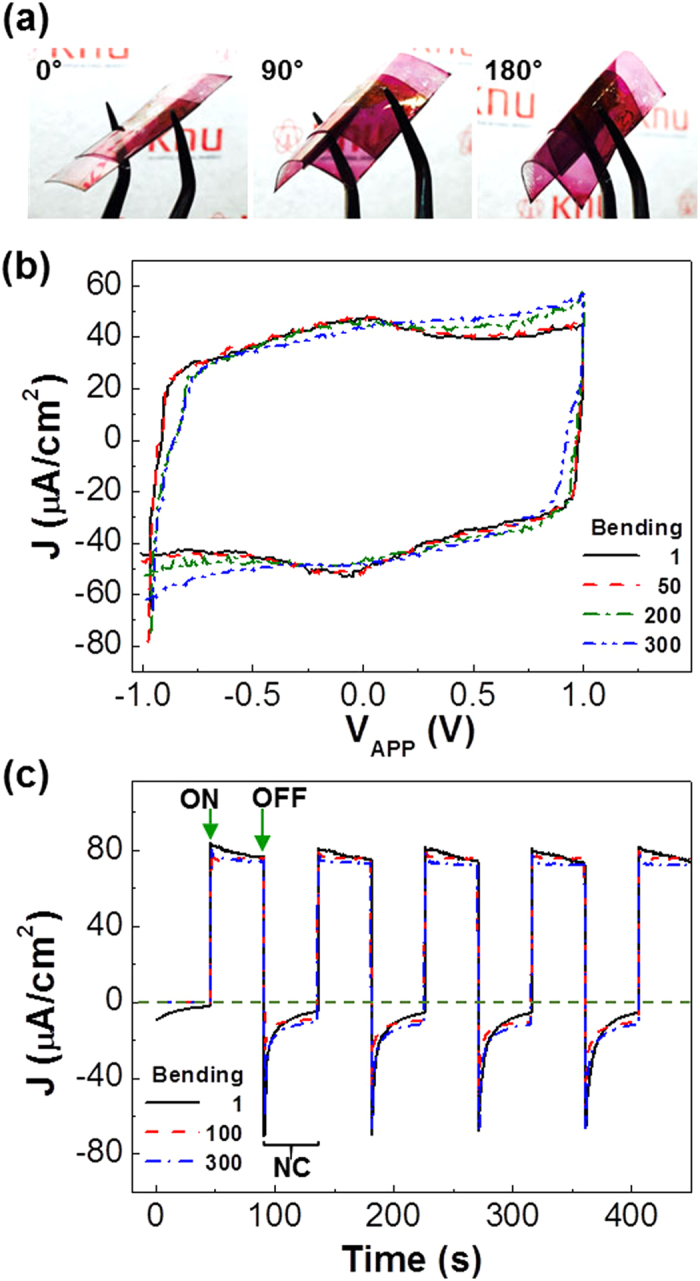
(**a**) Photographs for the flexible capacitors upon bending at a bending angle of 0°, 90° and 180°. (**b**) Cyclic voltammetry curves according to the number of bending (1, 50, 200 and 300 times) at an bending angle of 180°: The scan rate was 0.05 V/s. (**c**) Repeated potentiostatic curves for the flexible capacitors under illumination with a white light (100 mW/cm^2^) according to the number of bending (1, 100 and 300 times) at an bending angle of 180°: ‘ON’ and ‘OFF’ denote applying +1 V and removing +1 V (to 0 V), respectively.

Finally, the present flexible organic capacitors were subject to the bending test in order to investigate the device stability before and after bending. As shown in Fig. 7a, the flexile capacitors could be easily bent up to the bending angle of 180^o^ without any apparent damages. After bending for 300 times at 180^o^, the CV curves under the white light (scan rate = 0.2 V/s) were pretty well maintained in the presence of slight current increase in the edge parts (see Fig. 7b). In particular, as shown in Fig. 7c, the capability of photo-amplified charge storage under potentiostatic mode could be excellently kept even after device bending for 300 times at 180^o^. This result supports that the present flexible capacitors are quite stable upon bending under the white light and could amplify the charge storage by utilizing photons. In addition, the resulting flexible organic capacitors exhibited excellent cycling stability in the dark and light illumination conditions (see Fig. S5).

## Discussion

Flexible organic multilayer capacitors were fabricated by sandwiching the solid-state organic electrolyte (ALQSA_3_) layers between the light-sensitive conjugated polymer (P3HT, 50 nm) nanolayers coated on the ITO-coated PET film substrates. The fabricated flexible capacitors were optically semi-transparent enough for light penetration from both sides of the PET film substrates, while they were mechanically robust due to the good adhesion between the ALQSA_3_ layer and the P3HT nanolayers. A pronounced hysteresis was measured from the cyclic voltammetry measurements in the dark, which was well kept and even improved under illumination with a white light. The galvanostatic charging-discharging test revealed that the amount of charges (up to 1.5 V) stored in the present devices can be enhanced by increasing the charging time in the dark but the maximum voltage was decreased to 0.37 V by the white light illumination. Interestingly, the potentiostatic charging-discharging test disclosed that charges can be stored just after OFF state (0 V) due to the polarization effect by the transported proton ions, which could be remarkably (by ca. 250-fold) enhanced by the white light illumination. In particular, the bending test showed that the present flexible organic capacitors are considerably stable even after bending for 300 times at a bending angle of 180^o^. Hence it is expected that the present photo-amplification concept in capacitors may significantly contribute to the invention of brand-new category of energy storage devices.

## Methods

### Materials

The solid-state electrolyte material, tris(8-hydroxyquinoline-5-sulfonic acid) aluminum (ALQSA_3_), was synthesized with higher yield (>90%) by improving the previously reported method^28^. In brief, aluminum triisopropoxide (AltiP, 0.61 g, Sigma-Aldrich) and 8-hydroxyquinoline-5-sulfonic acid (HQSA, 2.03 g, Sigma-Aldrich) were mixed and dissolved in solvent (N,N-dimethylacetamide, 10 ml, Sigma-Aldrich). These reactant solutions were subjected to stirring for 10 min, followed by heating up to 100 °C. Then the condensation reaction was carried out at 100 °C for >6 h. After cooling for terminating reaction, the product solutions were poured into isopropyl alcohol (IPA) and resulting precipitates were purified by subsequent recrystallization processes. The finally purified ALQSA_3_ powders were dried in vacuum at 75 °C for 24 h. The ALQSA_3_ solution was prepared by dissolving the synthesized ALQSA_3_ powder in deionized water at a solid concentration of 50 mg/ml. The P3HT polymer (weight-average molecular weight = 30 kDa, polydispersity index = 1.7, regioregularity = 97%, Rieke Metals, USA) was dissolved in chlorobenzene at a solid concentration (20 mg/ml) leading to the P3HT solution. The ITO-coated PET films (thickness = 125 μm, sheet resistance = 30 Ω/□) were provided from Fine Chemical Co. (Republic of Korea).

### Film and Device Fabrication

Prior to device fabrication, the ITO-coated PET films were subject to photolithography/etching process in order to pattern the strip-type ITO electrodes (12 mm × 15 mm). The patterned ITO-coated PET substrates were cleaned with isopropyl alcohol using an ultrasonic cleaner. The cleaned substrates were finally dried with a nitrogen flow for quick removing of remnant liquids. Next, the P3HT nanolayers (thickness = 50 nm) were spin-coated on the ITO-coated PET substrates at 1500 rpm, followed by soft-baking at 60 °C for 15 min. The ALQSA_3_ films (thickness = 10 μm) were coated on the P3HT nanolayers and dried at 80 °C for 20 min. Another set of the P3HT nanolayer-coated ITO-PET substrates was stacked on top of the ALQSA_3_ film that was coated on the P3HT nanolayers, followed by post-treatment in vacuum at 75 °C for 24 h for complete adhesion between the ALQSA_3_ film and the P3HT nanolayers.

### Measurements

The optical absorption spectra of solutions and films were measured using a UV-visible spectrometer (Optizen 2120 UV, Mecasys), while a surface profiler (Alpha-step 200, Tencor) was employed for the film thickness measurement. The surface of films was examined with a scanning electron microscope (SEM, SU8220, Hitachi), while the apparent quality of films and devices was inspected with an optical microscope (SV-55, Sometech). The basic performance of capacitors was measured by employing either potentiostatic or galvanostatic mode using a multifunctional electrometer (Keithley 2636b). The optical charging experiment was carried out using the same electrometer and a solar simulator (100 mW/cm^2^, air mass 1.5 G filter, 92250A-1000, Newport Oriel). The adhesion test between the ALQSA_3_ layer and the P3HT nanolayer in the flexible organic capacitor was performed by fixing one side of the PET substrate to the laboratory stainless steel bar and by loading a water bottle (15 g) on another side of the PET substrate (note that Fig. 1d just shows no slip even by holding the device with fingers and moving/swing it).

## Additional Information

**How to cite this article**: Lee, H. *et al.* Strong Photo-Amplification Effects in Flexible Organic Capacitors with Small Molecular Solid-State Electrolyte Layers Sandwiched between Photo-Sensitive Conjugated Polymer Nanolayers. *Sci. Rep.* **6**, 19527; doi: 10.1038/srep19527 (2016).

## Supplementary Material

Supplementary Information

Supplementary Movie 1

Supplementary Movie 2
