# Supplementary material for: Strong Photo-Amplification Effects in Flexible Organic Capacitors with Small Molecular Solid-State Electrolyte Layers Sandwiched between Photo-Sensitive Conjugated Polymer Nanolayers
Source: Sci Rep. 2016 Feb 5;6:19527. doi: 10.1038/srep19527 (PMC4742829; doi:10.1038/srep19527)
Supplement: Supplementary Information [file srep19527-s1.pdf]

SUPPLEMENTARY INFORMATION

Strong Photo-Amplification Effects in Flexible Organic Capacitors with Small Molecular Solid-State Electrolyte Layers Sandwiched between Photo-Sensitive Conjugated Polymer Nanolayers

Hyena Lee,[1] Jungnam Kim[1], Hwajeong Kim[1,2], and Youngkyoo Kim[1]*

[1]Organic Nanoelectronics Laboratory, Department of Chemical Engineering, School of Applied Chemical Engineering, Kyungpook National University, Daegu 702-701, Republic of Korea.

[2]Priority Research Center, Research Institute of Advanced Energy Technology, Kyungpook National University, Daegu 702-701, Republic of Korea.

*Corresponding Author: ykimm@knu.ac.kr

This supplementary information has following data:

(1) **Figure S1**: Current density at 0 V as a function of scan rate.
(2) **Figure S2**: Enlarged current density – time plots from Figure 5 in the dark.
(3) **Figure S3**: Video images for the potentiostatic operation of flexible capacitors.
(4) **Figure S4**: Enlarged current density – time plots from Figure 6 in the dark.
(5) **Figure S5:** Cycling stability (potentiostatic mode) for flexible multilayer capacitors.

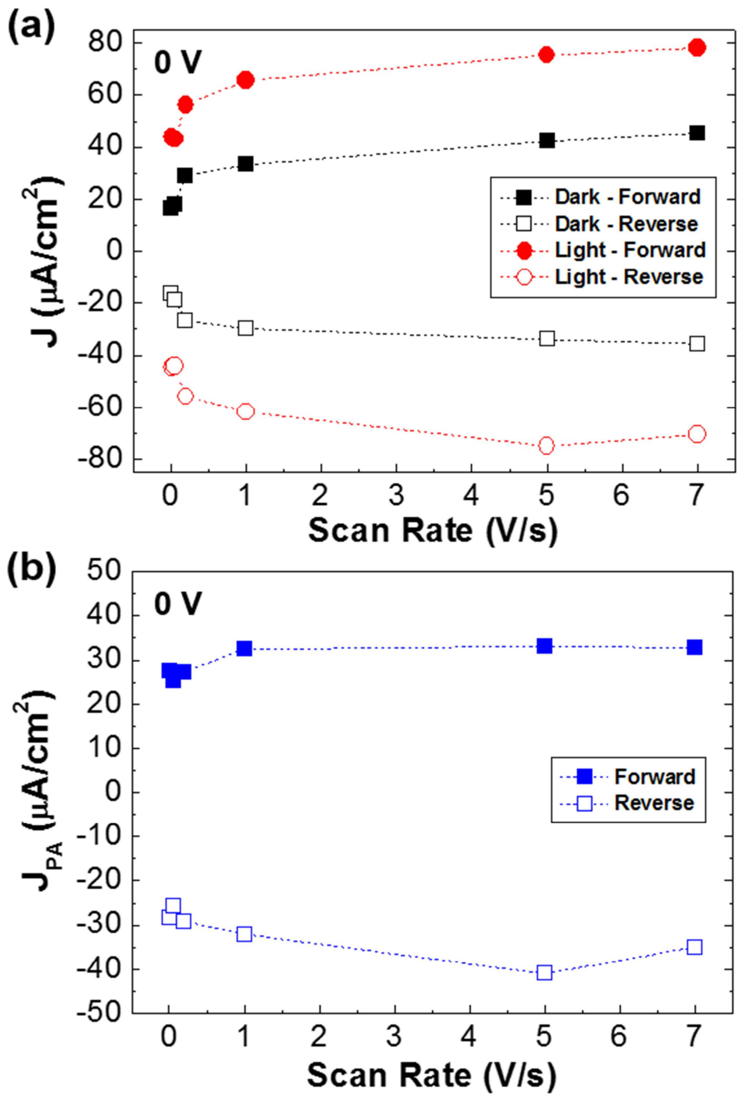

Figure S1. (a) Current density at 0 V as a function of scan rate in the dark and under illumination with a white light (data taken from Figure 1). (b) Net current density by photo-amplification at 0 V as a function of scan rate: The current density in the dark was removed from the current density under the white light in (a).

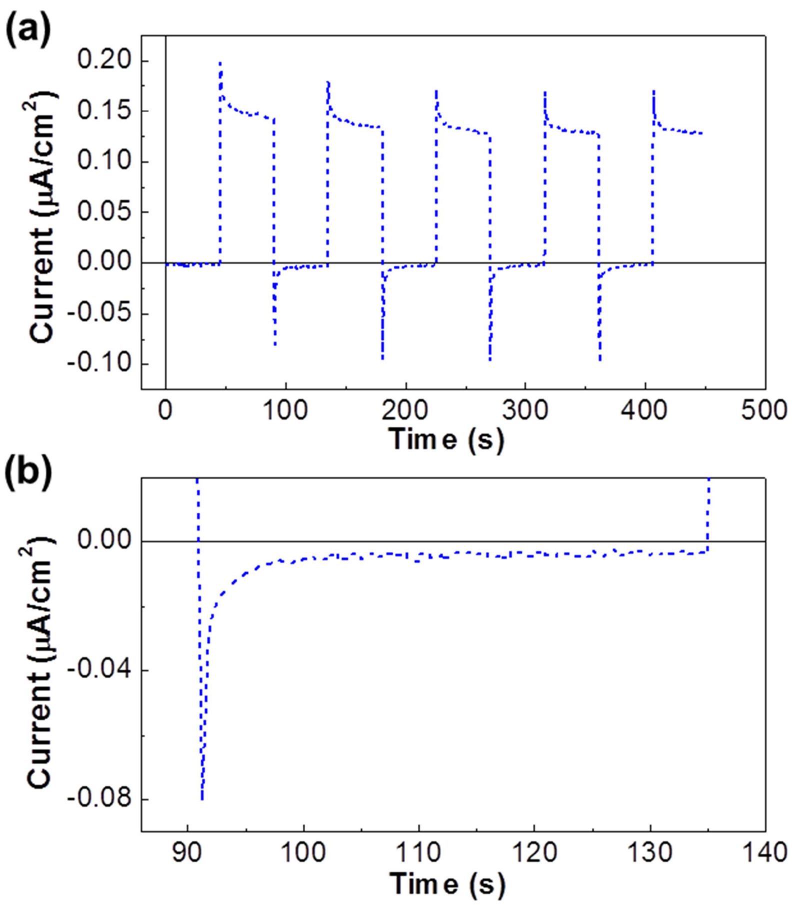

Figure S2. Enlarged current density – time plots from Figure 5 in the dark for better understanding: (a) from Figure 5(a) and (b) from Figure 5(b).

(a)

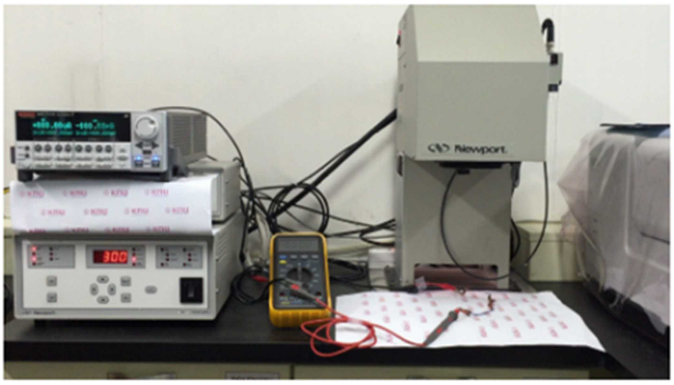

Operation in the Dark
(filename: DARK.avi)

(b)

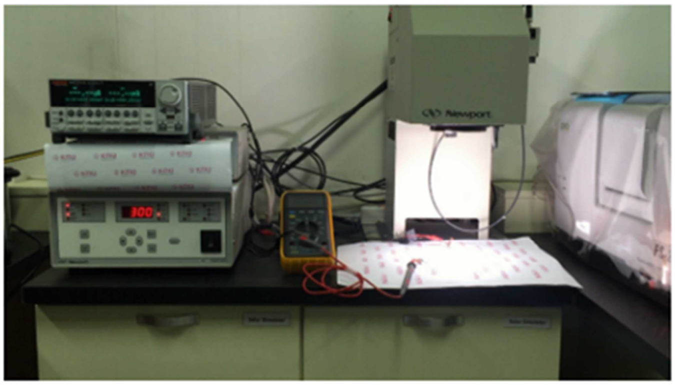

Operation Under White Light
(filename: LIGHT.avi)

Figure S3. Video images for the potentiostatic operation of flexible multilayer capacitors: (a) in the dark, (b) under illumination with a white (simulated solar) light (100 mW/cm^2). The operation was carried out by applying 0 V (OFF) and 1 V (ON) alternatively with an time interval of 11 s. The overall time was 90 s for the OFF state (a) and 120 s for the ON state (b), respectively (see attached video files).

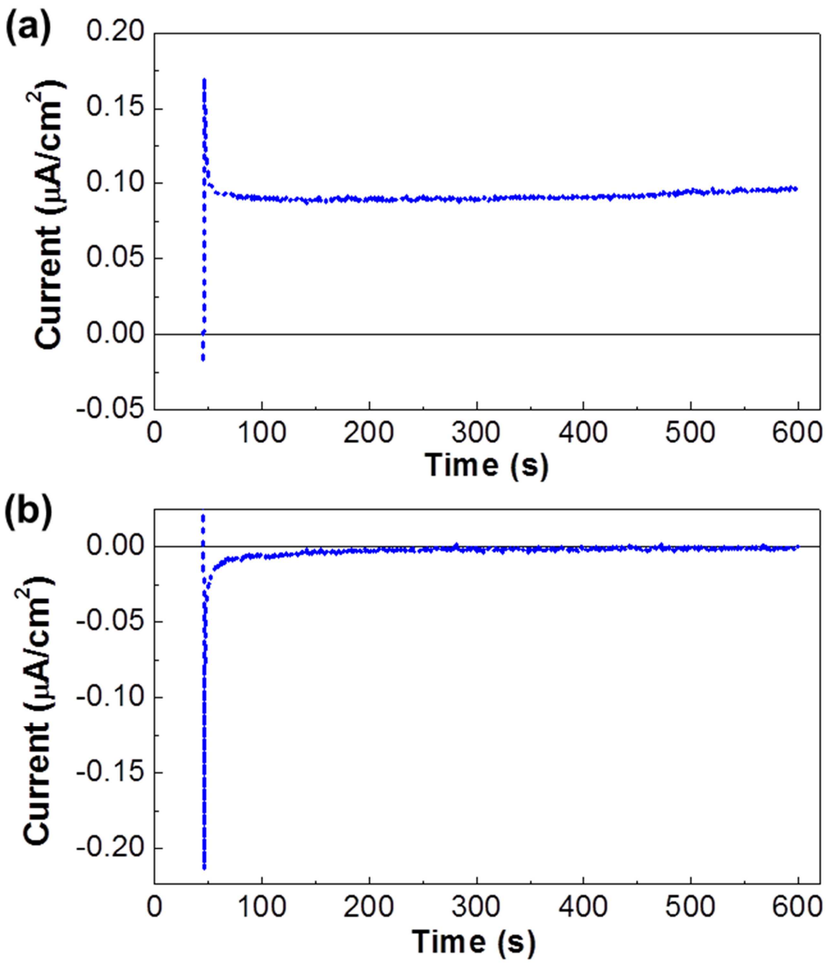

Figure S4. Enlarged current density – time plots from Figure 6 in the dark for better understanding: (a) from Figure 6(a) (1 V) and (b) from Figure 6(b) (0 V).

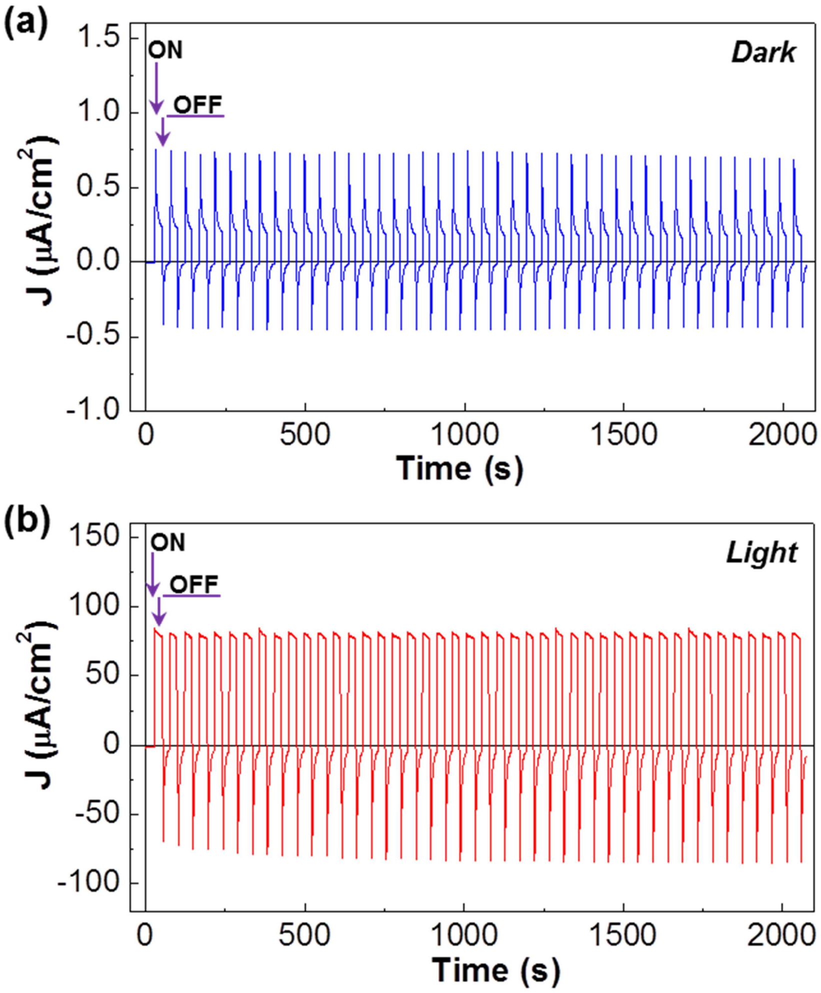

Figure S5. Cycling stability test result (potentiostatic mode) for flexible multilayer capacitors: (a) in the dark, (b) under illumination with a white light (100 mW/cm^2). The applied voltage condition was the same as in Figure 5. The stability test was carried out by applying the repetition of cycle program [ON at +1 V (20 s) ➔ OFF at 0 V (20 s)].
